# Lysosomal storage disorders: from biology to the clinic with reference to India

**DOI:** 10.1016/j.lansea.2022.100108

**Published:** 2022-11-21

**Authors:** Jayesh Sheth, Aadhira Nair, Babban Jee

**Affiliations:** a FRIGE's Institute of Human Genetics, FRIGE House, Jodhpur Gam Road, Satellite, Ahmedabad 380015, India; b Department of Health Research, Ministry of Health and Family Welfare, Government of India, 2nd Floor, IRCS Building, Red Cross Road, New Delhi 110001, India

**Keywords:** Lysosomes, Lysosomal storage disorders, Burden, Diagnosis, Prevention

## Abstract

Lysosomal storage disorders (LSDs) are a group of seventy different metabolic storage diseases due to accumulation of substrate mainly in the form of carbohydrate, lipids, proteins, and cellular debris. They occur due to variant in different genes that regulate lysosomal enzymes synthesis, transport, and secretion. In recent years, due to an increased availability of various therapies to treat these disorders, and increased diagnostic tools, there has been an escalated awareness of LSDs. Due to heterogeneous population and various social reasons, India is likely to have a high frequency of LSDs. Therefore, to understand the burden of various LSDs, its molecular spectrum, and understanding the phenotype–genotype correlation, Indian Council of Medical Research (ICMR) and Department of Health Research (DHR), Government of India had set up a task force in the year 2015. It has resulted in identifying common LSDs, and founder variant for some of the storage disorders and molecular spectrum of various LSDs across the country. This review describes in detail the spectrum of LSDs, its molecular epidemiology and prevention in context to Indian population.

## Introduction

Lysosomal Storage Disorders (LSDs) are a group of more than 70 inherited metabolic diseases arising due to defects in genes encoding lysosomal proteins. The majority of these proteins are lysosomal hydrolases, accessory proteins, membrane transporters, or trafficking proteins.^1^ The primary function of lysosomes is to maintain the healthy cellular process by its involvement in autophagy, cholesterol homeostasis, and repair of the plasma membrane, bone remodelling, defence against pathogens, cell death and signalling. Hence, deficiency of lysosomal proteins causes unwanted accumulation of biomolecules inside the lysosomes, ultimately resulting in cell dysfunction and death. These observations to some extent have helped in the understanding of complex pathophysiology of LSDs.^2^

**Fig. 1:**
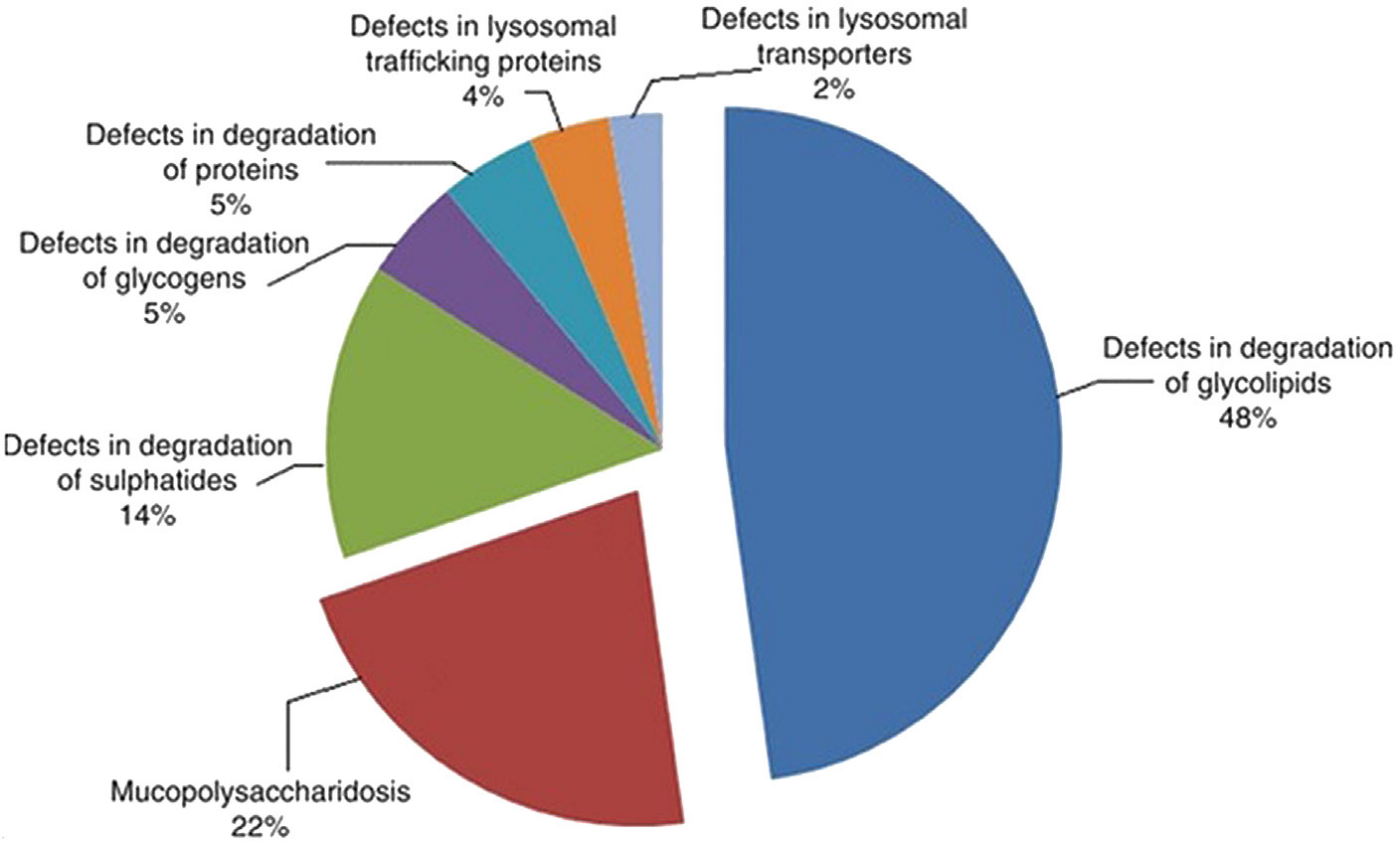
Distribution of different storage disorders in India (Sheth et al., 2004 and 2014).

LSDs are classified based on the type of storage material into sub-categories namely: sphingolipidoses, mucopolysaccharidoses, glycoproteinoses, lipid storage disease, and glycogen storage disease. Also, based on the age of onset of initial symptoms, they are classed into infantile, juvenile, and adult-onset forms. The combined incidence of LSDs worldwide is ∼1 in 5000–7500 births with a higher incidence of certain LSDs in specific population.^1^ For instance, the Ashkenazi Jewish population has a high frequency of 1 in 855 births for Gaucher disease.^3^ Likewise, neuronal ceroid lipofuscinoses (NCL) have a higher incidence in Finland.^4^ The phenomenon of a founder effect and genetic isolation in addition to the practice of consanguinity are the possible explanations for the observed high incidence of certain LSDs. Likewise, in India, Sheth and his group described the burden of LSDs (Fig. 1).^5^^,^^6^ Their study also demonstrated the presence of a founder variant for Tay-Sachs and Morquio-A disease in a particular community of Gujarat, India.^6^^,^^7^ They have also observed a common variant L444P in the *GBA1* gene for Gaucher disease patients pan India.^8^

LSDs are multi-systemic disorders with involvement of the nervous system in ∼70% of the cases.^9^ The key initial noticeable features in most patients include coarse facies, regression in learned skill, hepatosplenomegaly, respiratory distress, skeletal dysplasia, presence of cherry red spot and neurodegeneration.^5^^,^^6^^,^^9^ Nonetheless, there is a clinical and genetic heterogeneity seen in patients of LSDs. Several factors such as the type of underlying variant and the amount/activity of residual mutant protein are likely to be associated with the severity of clinical presentation in these patients. Also, the effect of gene variant on the protein activity and the resulting disease course is not well understood in the case of all LSDs. In addition, there are plausible studies to explain the phenotype variability seen in LSD patients harbouring the same variant. The role of other factors such as epigenetic factors, the effect of modifier genes, and environmental factors on clinical heterogeneity remains unclear.^1^

### History of LSDs in India

**Fig. 2:**
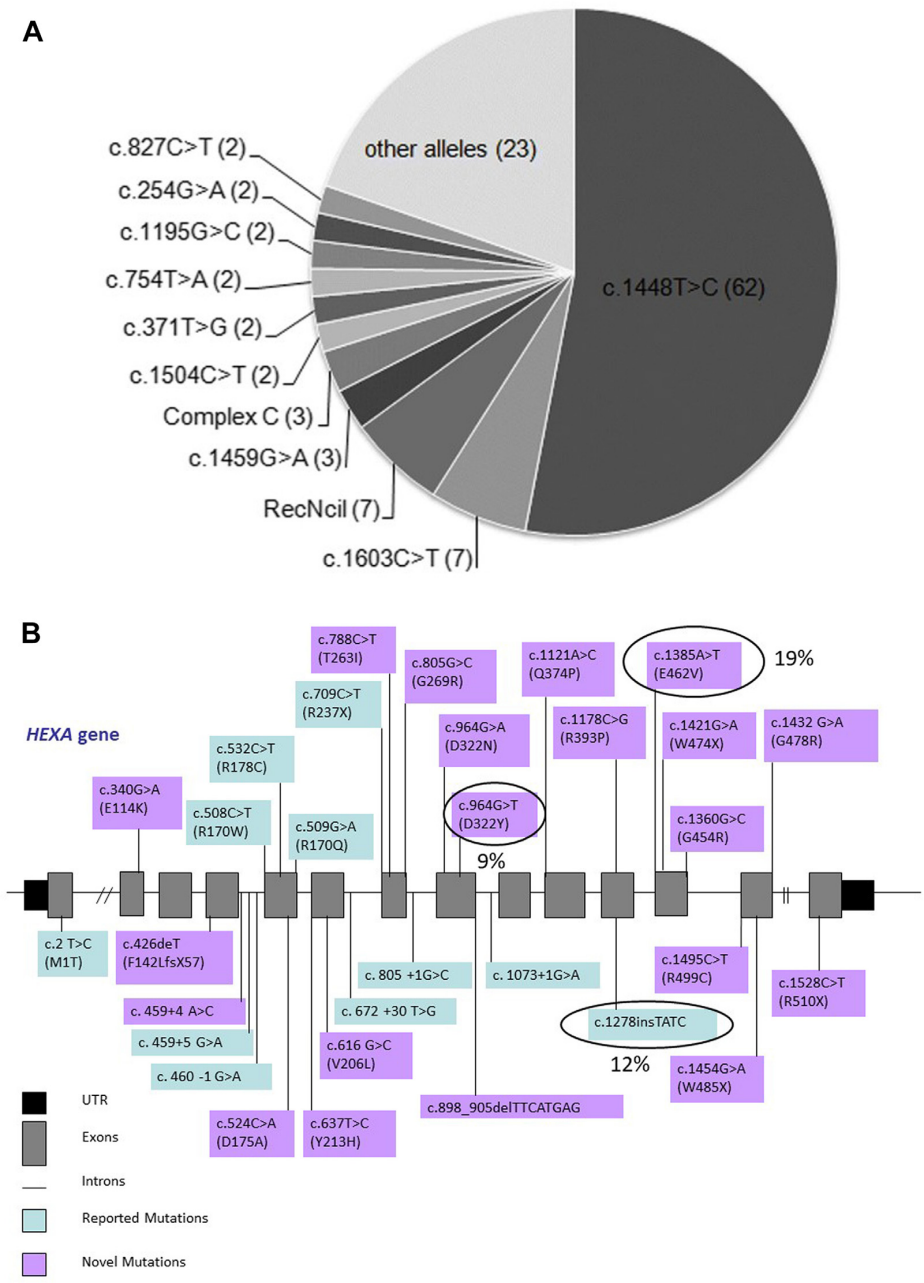

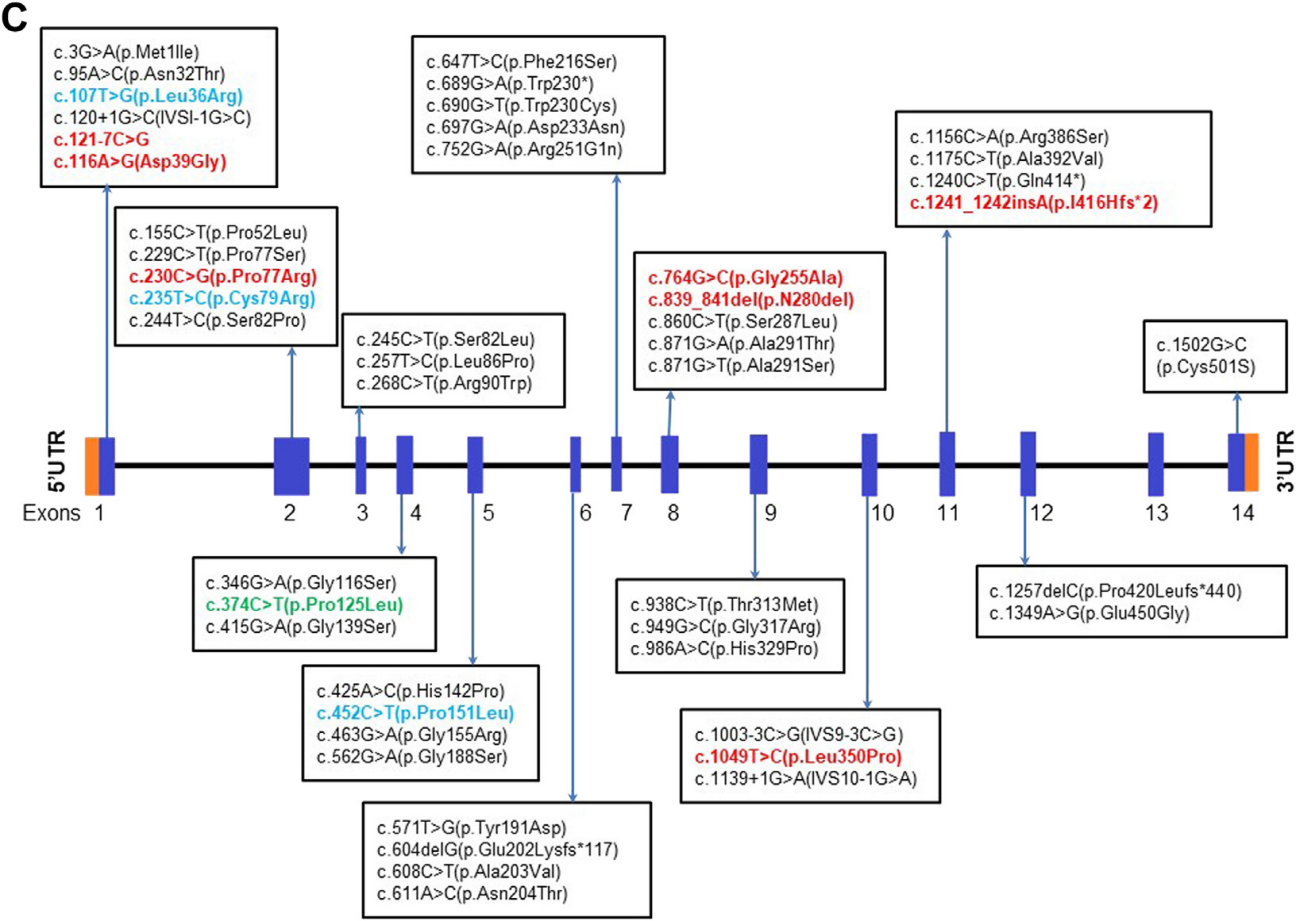
A: Illustrative representation of the distribution of the variants identified in Indian patients with Gaucher disease (Sheth et al., 2019). B: Illustrative representation of the distributions of the variants identified in Indian patients with Tay-Sachs disease (Sheth et al., 2014). C: Schematic representation of variants identified in the *GALNS* gene in MPS IVA patients from India (Sheth et al., 2022).

Pompe disease was the first LSD described by Henri G Hers in 1963. Following this, several studies between 1960 and 1980 further characterised and identified many LSDs. In India, there has been a lack of awareness about this group of genetic disorders. A very few independent case reports for LSDs were reported in India between 1970 and 2000. A large study including 18 mucopolysaccharidosis (MPS) patients was reported in 1986 from Calcutta.^10^ Later in 1988, another group from G. S. Medical College and KEM Hospital, Mumbai; described a large cohort of 48 MPS cases.^11^ Interestingly, in the last two decades, there has been a considerable contribution to understand the burden of LSDs in India. Also, several studies have elucidated the variant spectrum of a few LSDs like Gaucher disease (Fig. 2A), Tay-Sachs disease (Fig. 2B), Batten disease, Niemann-Pick disease, Morquio-A disease (Fig. 2C), Mucolipidosis III alpha, beta and gamma,^12^^,^^13^ and MPS II.^14^ The first attempt to treat this group of disorders using enzyme replacement therapy (ERT) was made in 1990.^15^ In India, Gaucher disease was the first LSD to be treated with ERT in 1999.^16^

### Burden of LSDs in India

**Fig. 3:**
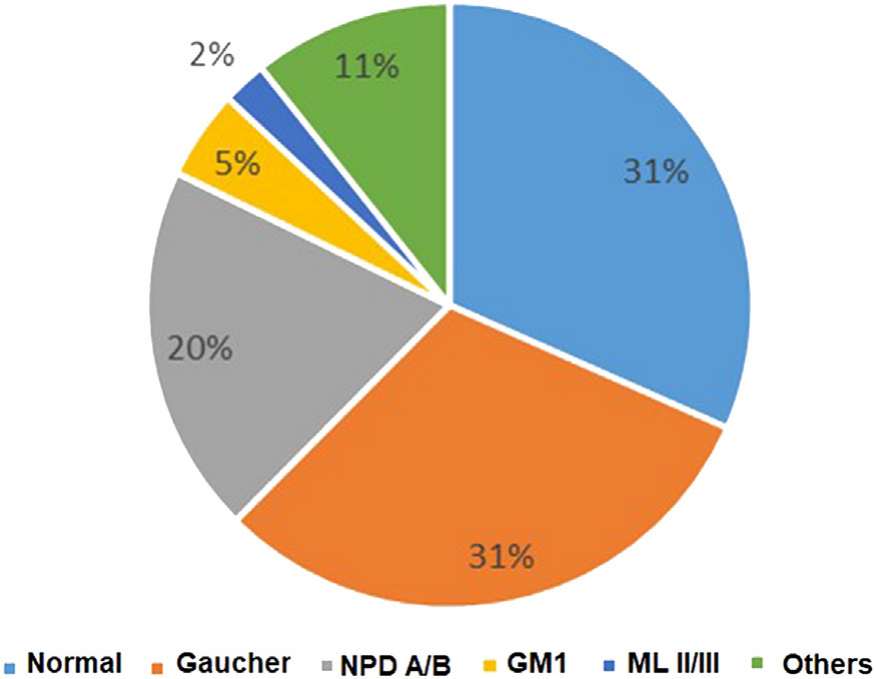
Distribution of various LSDs among children with Hepatosplenomegaly (N = 253).

Various studies have addressed the burden of LSDs in India. Sheth and his group for the first time reported the prevalence of different LSDs in a cohort of 150 children^5^ and 387 children^6^ with high clinical suspicion of LSD. They found glycolipid storage disorders (48%) to be the most common LSD followed by the MPS group (22%). Among the glycolipid storage disorders, Gaucher disease was the most common disease reported. This observation is similar to another study reported by Verma et al.^17^ in 2012, in the cohort of LSD patients from North India. Also, Agarwal et al.^18^ in their study cohort showed highest percentage of Gaucher disease patients (32%) followed by MPS disorder (20%). A recent study by Goyal and Gupta^19^ in a cohort of 69 LSD children also reported a high proportion of Gaucher disease patients. In another study carried out by Sheth et al. in children presenting with hepatosplenomegaly, anaemia and thrombocytopenia, highest number of children were diagnosed with Gaucher disease followed by other LSDs (Fig. 3). Sheth et al. have also shown a higher incidence of Tay-Sachs disease which is possibly due to the presence of a founder variant in the *HEXA* gene^20^^,^^21^ in the Parmar community of Gujarat originating from Saurashtra region. Likewise, a study by Nalini and Christopher has reported a high prevalence of GM2 gangliosidosis in patients from southern India.^22^

MPS disorders are the second most commonly reported LSDs in India.^6^ Amongst these, MPS II (39%) was reported to be the most prevalent followed by MPS I (30%) and MPS IVA (17%).^19^ A recent study by Sheth and his group has shown the presence of a founder variant P77R in the *GALNS* gene in MPS IVA patients from Gujarat in a particular ethnicity.^7^ Another group of LSDs consisting of Batten disease, Pompe disease, and Fabry disease has a low prevalence in the country. However, in absence of accurate data, the observed low percentages may be due to lack of awareness among clinicians and a dearth of diagnostic facilities across the country.

Interestingly, a couple of rare LSDs have also been reported from India. Niemann Pick type C is a clinically heterogeneous condition caused due to variant in *NPC1* or *NPC2* genes. A study by Sheth et al.,^23^ 2017 demonstrated that NPC2 can present in the early years of life with pulmonary complications like alveolar proteinosis and hepatosplenomegaly or hepatomegaly. Early suspicion is critical in such cases for the management of the condition. GM2 gangliosidosis AB variant is another rare neurodegenerative disorder caused due to deficiency of GM2 activator protein. There are only 12 cases reported in the literature, that include two cases from India with a novel variant in the *GM2A* gene.^24^^,^^25^

Scarce data is available on the adult-onset LSDs in India. One study was carried out by Sheth et al.^26^ on adult Gaucher patients. Several other studies such as a single case of Juvenile onset GM2 gangliosidosis,^27^ two cases of Late infantile form of multiple sulphatase deficiency (BMC Pediatrics, 2022 under review), and adult onset Sialidosis in a 24 years old male with progressive myoclonic ataxia due to variant in the *NEU1* gene (personal communication Sheth et al., 2022) suggest that there are adult forms of LSDs in the country and they might be underdiagnosed due to poor awareness.

All these studies suggest the need to prepare an algorithm for an early clinical suspicion of LSDs, its timely referral to a tertiary genetic centre followed by accurate genetic testing to identify such rare cases of LSDs in the country.

### Diagnosis of LSDs in India

**Fig. 4:**
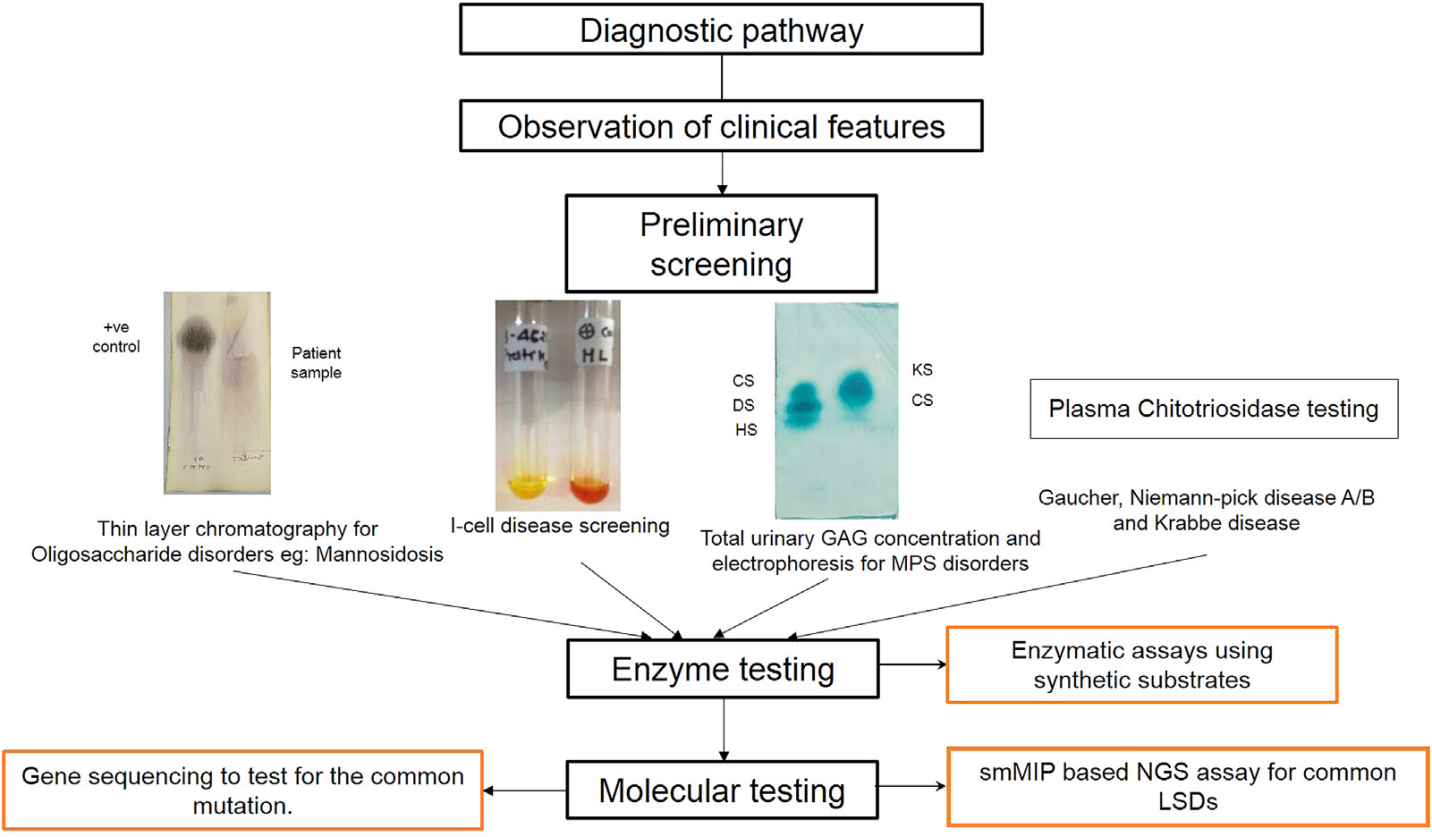
Diagnostic algorithm for LSDs.

Early diagnosis is the most critical part of the management of LSDs as it provides the opportunity for therapeutic intervention, precise genetic counselling, prenatal diagnosis and a better outcome for the patient and relief for the family members. The diagnosis of LSDs in India currently involves a three-step approach. The preliminary key step is noting the presenting clinical indications, and based on this, an initial LSD suspicion is made, followed by a screening test. There are number of effective screening strategies available for different classes of LSDs. Like in the case of MPS disorders, a urine glycosaminoglycan (GAG) test is widely used. Following this, qualitative urinary GAG analysis is performed. The basic principle of these tests is to look for the presence of different GAGs like keratan sulfate (KS), dermatan sulfate (DS), heparan sulfate (HS), and chondroitin sulfate (CS) (Fig. 4). In our experience, the quantitative and qualitative urinary GAG analysis test is 100% sensitive and 68% specific and should be used routinely as a first-tier study for suspected MPS patients and this will also considerably reduce the number of individuals requiring additional tests and/or investigations. Patients with clinical suspicion of MPS and negative GAG screening should be tested for atypical oligosaccharide pattern. Another routinely used screening test is chitotriosidase testing as a biomarker for Gaucher and Niemann Pick A/B diseases (NPD A/B). In India, Sheth et al. studied the significance of chitotriosidase testing in patients presenting with hepatosplenomegaly, thrombocytopenia and anaemia (Fig. 4). Also, chitotriosidase screening test is highly specific for the diagnosis of Gaucher disease and Niemann-pick disease A/B.^28^ Absent or low activity of Chitotriosidase has also been observed in around 5% of cases with Gaucher disease in our study which could be due to presence of a null allele of Chitin gene. Although, we have not carried out molecular study to identify the common 24 base pair duplication in *CHIT1* gene which has been observed in European population.^29^ Recently, a study by Kadali et al.^30^ in 2016 showed 84% sensitivity and 100% specificity of chitotriosidase screening test for Gaucher disease and a 72% sensitivity and 84% specificity for NPD. Likewise, we have demonstrated the use of a simple chemical screening method for I-cell disease. It is 100% sensitive and specific.^31^ In case of oligosaccharidosis (subgroup of LSD caused due to deficient glycoprotein degradation), there is an excess excretion of urine oligosaccharides. Thin-layer chromatography screening method is widely used for the rapid identification and characterization of these diseases.^32^ Multiplex screening test for 25 sulfatiduria associated LSDs has been shown to be useful for the initial screening by tandem mass spectroscopy.^33^

Recent studies have shown the importance of Lyso Gb1 and Lyso Gb3 as an useful biomarker for monitoring the treatment of patients with Gaucher and Fabry disease respectively.^34^^,^^35^

Although there are reliable screening tests available for different LSDs, confirmatory tests are essential for accurate diagnosis and management of the condition. These include enzyme study from leucocytes and molecular study. Enzyme testing involves measuring levels of lysosomal enzymes in dried blood spots (DBS) or leukocytes isolated from a blood sample using 4-Mu fluorogenic synthetic substrates. However, for LSDs due to deficiency of activator protein, assessing enzyme levels will not be informative. Enzyme testing have wide clinical utility and hence despite the pitfalls, it is considered as the gold standard for the diagnosis of LSDs.^1^

A molecular study is preferable for confirming the diagnosis of LSDs that can help in identifying the carrier status in other unaffected members in the family. Additionally, for prenatal diagnosis, molecular study is the gold standard than carrying out enzymes study from the cultured chorionic villi or amniotic fluid fibroblasts, as the latter require more expertise, standardisation, and skilled scientists. In cases like the Gaucher disease, genotype identification also helps in defining the neuropathic form from a non-neuropathic or sub-acute neuropathic form of the disease. Likewise, genetic tests are obligatory in case of inconclusive biochemical results.

Several approaches including Sanger sequencing and the use of next generation sequencing (NGS) technologies have elucidated the variant spectrum of different LSDs. Table 1 enlists the common variants observed in different LSDs in the Indian population. This information is noteworthy as a targeted analysis of the corresponding gene can be carried out as a first-line test for variant detection in suspected LSD cases.

**Table 1:** Common variants in different LSDs across the Indian population.

Disease	Common variant	Subjects	Region	Reference
Gaucher disease	L444P (c.1448T>C)	21/33 patients	Pan-India	Ankleshwaria et al. 2014^36^
	L444P (c.1448T>C) R535C (c.1603C>T)	100 patients 60% 7%	Pan-India	Sheth et al. 2019^8^ (DHR-ICMR-NTF-LSD)
Niemann Pick disease A/B	p.R542∗ p.R418∗	60 families 21.67% 6.6%	Pan-India	Ranganath et al. 2016^37^ (DHR-ICMR-NTF-LSD)
	p.R542∗	3/40 patients (7.5%)	Pan-India	Deshpande et al. 2021^38^
MLD	c.931C>T	14/122 alleles	Pan-India	Narayanan et al. 2018^39^
GM1 gangliosidosis	c.75+2InsT	50 families 14% (allele frequency)	Pan-India	Bidchol et al. 2015^40^
MPS I	p.Arg619∗ p.Ala75Thr	30 patients 25% 20%	Pan-India	Uttarilli et al. 2016^41^
MPS II	p.Gly374sp p.Arg88His	30 patients 40% 20%	Pan-India	Uttarilli et al. 2016^41^
	p.Arg88His	6/71 patients (8.4%)	Pan-India	Agrawal et al. 2022^14^ (DHR-ICMR-NTF-LSD)
MPS IVA	p.Ser287Leu p.Phe216Ser p.Asn32Thr p.Ala291Ser	68 families 8.82% 7.35% 6.61% 5.88%	Northern and Southern part of India	Bidchol et al. 2014^42^
	p.Pro77Arg	14/23 families	Gujarat	Sheth et al. 2022^7^ (DHR-ICMR-NTF-LSD)
MPS VI	p.W450C (c.1350 G>C) p.L98R: Founder variant	4/14 families 2/8 families	Pan-India South-India	Uttarilli et al. 2015^43^ Mathew et al. 2015^44^
Tay-Sachs disease	p.E462V c.1278insTATC	6/15 families 7/34 patients	Gujarat Pan-India	Mistri et al., 2012^20^ Sheth et al. 2014^2^^1^ (DHR-ICMR-NTF-LSD)
Sandhoff disease	p.R284X	4/19 patients	Pan-India	Tamhankar et al. 2016^45^
Mucolipidosis II/III	c.3503_3504delTC	64 patients 28.35% (allele frequency)	Pan-India	Pasumarthi et al. 2020^13^
Pompe disease (Infantile onset)	c.1942G>A (p.Gly648Ser) c.1A>G (p.Met1?) c.2783A>G (p.Tyr928Cys) c.1003G>A (p.Gly335Arg)	10/96 alleles 8/96 alleles 7/96 alleles	Pan-India	Gupta et al. 2020^46^
Batten disease (NCL-I)	c.713C>T (p.Pro238Leu)	12 patients 44%	Southern India	Sheth et al. 2018^47^ (DHR-ICMR-NTF-LSD)
Batten disease (NCL-II)	c.616C>T (p.Arg206Cys)	22 patients 26%	Southern India	Sheth et al. 2018^47^ (DHR-ICMR-NTF-LSD)

∗ DHR-ICMR-NTF-LSD: Indian Council of Medical Research-Department of Health Research-National Task Force for Lysosomal storage disorders.

Interestingly, the last decade has seen an exponential advancement in the field of genomics and its technologies. It has led to the increased application of targeted gene panel-based sequencing approaches in the clinical diagnosis of LSDs. It is a hypothesis-free approach, looking at the genetic architecture of the patients and detecting pathogenic variants based on deep phenotyping. One of the latest high-throughput technologies is the use of single-molecule molecular inversion probes (smMIP) to design a target gene panel. The smMIP-based NGS approach provides a potentially flexible and affordable method for variant detection, both single nucleotide variants and copy number variants, thereby obliviating the need for MLPA.^48^

Despite the existing diagnostic setup, there are several challenges in achieving an accurate and timely diagnosis of LSDs in India. A study by Agarwal et al.^18^ showed an alarmingly high dropout rate of 62% of the 532 patients referred to their clinic with an LSD suspicion. In 60% of the cases, the patients had to travel from outstation. The above mentioned observation suggest that there is dearth of diagnostic facilities in our country. In such a scenario, setting up facilities that can provide simple screening tests can be one of the strategies for the early diagnosis of suspected patients. The major hurdle is the paucity of quality assured labs in the country. Additionally, there is a serious lack of awareness among physicians. A study by Glass et al. in Toronto highlights the importance of awareness of LSDs among clinicians. They stated that 81% of the cases were diagnosed within 1 month of presentation to the metabolic specialist because at least 45% of the referring physicians had initiated the diagnostic investigations for the same.^49^ In India, Agarwal et al.^18^ showed 14 months as the median time to reach a final diagnosis after the disease onset. In another study by Nampoothiri et al.^50^, a delay of 11.7 years in the confirmation of the diagnosis of Fabry disease from the time of initial presentation was reported. One of the contributing factors could be poor physician awareness as well as the limited resources in the country to set up specialised genetic centres. In India there are about five centres (FRIGE's Institute of Human Genetics in Ahmedabad, Sir Gangaram hospital in New Delhi, Centre for DNA finger printing in Hyderabad, Sandor Laboratory in Hyderabad and SGPGI in Lucknow) that provide enzyme testing facility while molecular testing for LSDs is widely available in the country. There is also a DISHA program run by Sanofi-Genzyme in collaboration with SGRH in Delhi and FRIGE in Ahmedabad to screen children with five common LSDs namely Gaucher, Niemann Pick-A and B, Pompe, Fabry and Hurler disease free of cost.

**Fig. 5:**
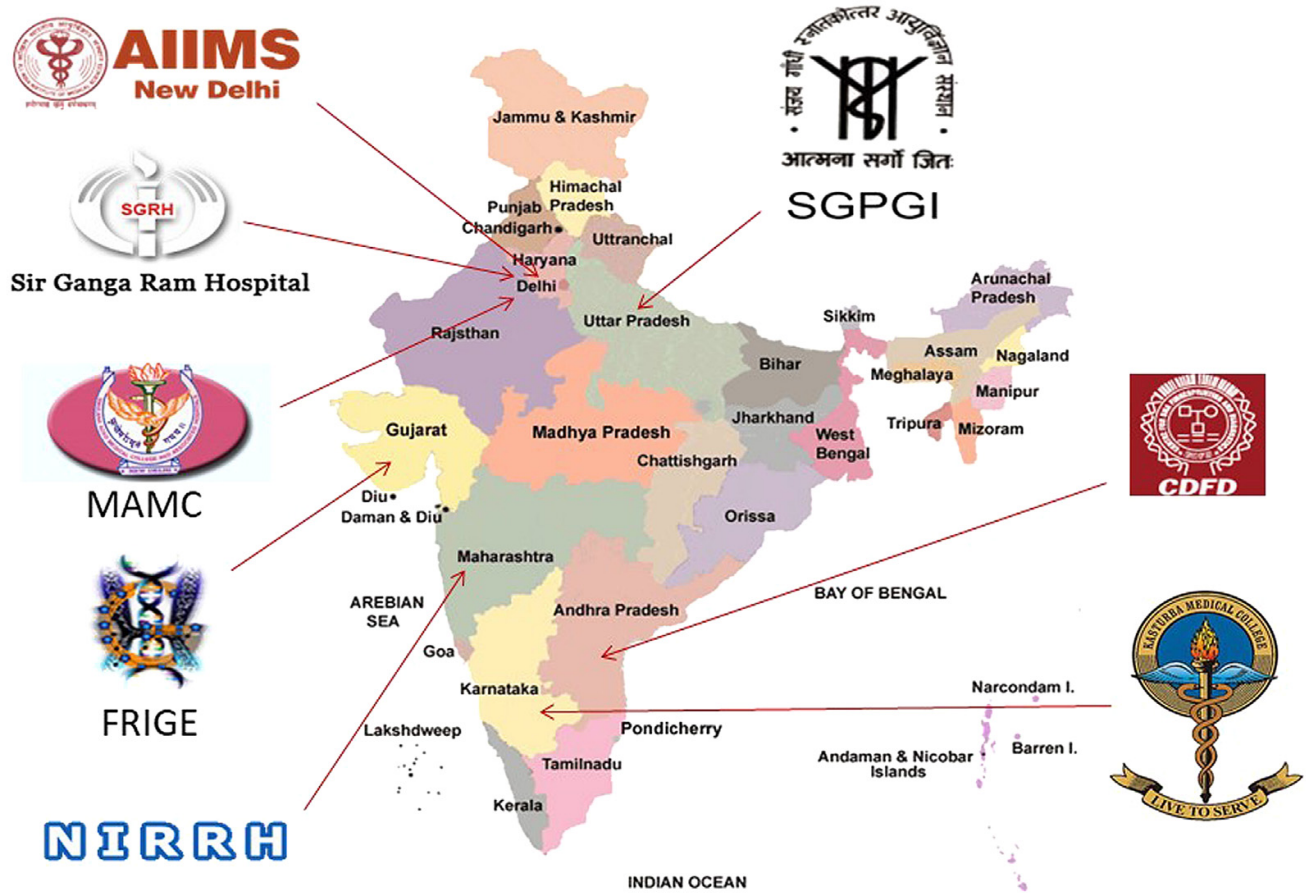
Collaborating centres in the DHR-ICMR funded study on “Clinical, biochemical and molecular characterization of Lysosomal storage disorders in India.

To identify the burden and spectrum of LSDs, and understand the pathophysiology in Indian context, Indian Council of Medical Research (ICMR) set up a special task force on LSDs from 2015 to 2018 involving eight centres across the country each, studying specific LSD (Fig. 5). This has made it possible to understand the variable disease burden in many parts of the country as shown in Table 1.

### Training programmes for LSDs

Anecdotal evidence suggests that awareness of diagnostic features, classification, and the utility of genetics in LSDs is limited in the Indian healthcare system. This is in part exacerbated by minimal introduction of biochemical genetics amongst medical and biomedical higher educational settings in India. To the best of our knowledge, there are two national fellowship programmes for clinical genetics and rare diseases of which, one is funded by the ICMR and organised by the Sanjay Gandhi Post Graduate Institute at Lucknow and the second is Sanofi-Genzyme Fellowship program in clinical genetics which is carried out at 7 centres across the country. With an ever increasing in demand as well as introduction of such programmes at new institutions across India, it is anticipated that it would have an impact on identification, diagnosis, treatment, and prevention of LSDs.

## Clinical, biochemical, and molecular profile of common LSDs in India

### Gaucher disease

It is one of the most common LSDs in the world with an incidence of 1 in 40,000–60,000 live births and a high carrier frequency of 1 in 14, particularly in the Ashkenazi Jewish population.^51^ It is also a common LSD in India as reported by several groups,^18^^,^^36^ however, the precise incidence is still unknown. The common presenting symptoms in Gaucher patients include hepatosplenomegaly, cytopenia, and anaemia. Based on the presence or absence of neurological manifestations, these patients are classified into types I to III. The early signs appear from infancy to late childhood with a median age of 3.6 years.^52^ The severe form of Gaucher disease is seen in type II where the disease progression is rapid and patients generally die by the age of 2 years.^51^ In India, as L444P genotype is seen in almost 60% of the Gaucher patients, Type III Gaucher disease can be considered more common than that in the Western population. Nonetheless, these patients are presented without neuronopathic involvement initially which may progress to subacute neuronopathic form, but this needs a larger cohort and longer follow up of the patients. As per personal communication with Dr Ashish Bavdekar from KEM Hospital, Pune and Dr Mamta Muranjan, from KEM Hospital, Mumbai, nearly 70–90% of children with L444P mutation progress to type III Gaucher and as per INCAP program, all children receiving ERT with L444P mutation have developed type III Gaucher disease over the span of nearly 20 years. The earliest and most common neurological manifestation in these patients includes oculomotor apraxia.^51^ In India, children with GD are presented with severe phenotype of massive splenomegaly and moderate hepatomegaly, while in adult GD patients, there is mild hepatosplenomegaly with or without bone involvement.^26^

Checking the levels of enzyme glucocerebrosidase in blood leukocytes is the gold standard test for the diagnosis of Gaucher patients. However, it is critical to note that false-positive results have been seen in cases where the differential diagnosis was Niemann-Pick type C, which could be due to sphingolipid interference in enzyme activity from leucocytes (personal communication with Jayesh sheth and group). Also, in rare cases, atypical Gaucher disease is due to saposin C deficiency (transporter protein), which results from biallelic variants in the *PSAP* gene, enzyme estimation in blood might not be informative. A single case of acute Gaucher Disease-Like Condition in an Indian Infant with novel biallelic variant in the Prosaposin Gene was recently reported.^53^ Hence, genetic diagnosis is critical in such cases.

Molecular diagnosis is, therefore, crucial to confirm the diagnosis, and to perform carrier testing and prenatal diagnosis. There are more than 300 variants reported in the *GBA1* gene known to be associated with Gaucher disease, of which L444P (c.1448T>C) is the most prevalent in India.^36^ Gaucher patients homozygous for c.1448T>C variant generally have a severe phenotype with nervous system involvement. However, in India, there is an extreme variation in phenotype of Gaucher patients with homozygous variant c.1448T>C which ranges from lethal collodion skin baby at birth to a less severe type III phenotype to a non-neuropathic form of the disease as seen in type 1.^54^

### Mucopolysaccharidosis (MPS)

MPS class of disorders are the second most common LSDs in the country as reported by several groups.^6^^,^^19^ Overall, there are twelve types of MPS disorders caused due to the deficiency of lysosomal enzymes involved in the degradation of glycosaminoglycans (GAGs).^1^ GAGs belong to the family of complex linear polysaccharides, composed of repeating disaccharide units. GAGs are present in all mammalian tissues and were initially known to have a key role in cell hydration and structural scaffolding. Recent studies however depict GAGs to be involved in cell signalling and they modulate several biochemical processes that are fundamental for cell biology, including regulation of cell growth and proliferation, promotion of cell adhesion, anticoagulation, wound repair, and others.^55^ Thus, any defect in the degradation of these molecules is highly likely to have serious consequences, with multisystem involvement.^56^ The common presenting symptoms include a large head, coarse facies, short stature, skeletal abnormalities, and hepatosplenomegaly.^57^ Several factors contribute to the delay in the early diagnosis of MPS disorders. These include variability in age and severity of manifestations, the attenuated phenotype in some cases, and a wide range of symptoms. An observational study by Grewal and Muranjan over a period of 11 years at a genetic clinic in Mumbai showed that out of 114 confirmed LSD patients, 35% contributed to MPS disorders.^58^ They reported a median time lag of 35.8 months between the onset of symptoms and diagnosis. They stated the common causes of delayed diagnosis to be a) symptoms overlooked by a physician, b) late medical consultation by a caregiver, and c) resource limitation. In 45% of the diagnosed MPS cases, the clinician had initially suspected an alternate clinical condition. In their study, 50% of the MPS IVA patients were misdiagnosed as rickets, skeletal dysplasia, and achondroplasia. Likewise, MPS I may be mistaken for rickets or rheumatoid arthritis and cases of MPS III may be mislabelled as a case of autism or attention deficit hyperactivity disorder. Hence, geneticists and metabolic specialists play a key role in making timely correct diagnoses of LSDs.

Though in country like India, there is no dearth of rare LSDs, region wise prevalence of these disorders is poorly understood except for few, like, Tay Sachs and Morquio-A in Gujarat due to founder variant. Likewise, Fabry and Pompe disease is commonly seen in Southern part of the country. The high prevalence of some LSDs could be due to increased awareness among the clinicians or due to practice of consanguinity in these communities, however, the exact reason remains unknown.

## Treatment for LSDs in India

Considering the clinical and genetic heterogeneity seen in LSDs, there have been great challenges in establishing therapeutic approaches for LSDs. Nevertheless, with continuous efforts and advancements in this area, a number of strategies have been developed. These include enzyme replacement therapy (ERT), hematopoietic stem cell therapy (HSCT), substrate reduction therapy, the use of chaperones, and gene therapy. It is important to know that these options do not cure the patient but slow the disease progression.^59^ ERT involves compensating for the deficient enzyme by administering recombinant enzymes. ERT has been a successful treatment approach in the case of Gaucher disease in India, as shown by a retrospective review by Nagral et al.^52^ At present, recombinant ERT is available in India for Gaucher's disease (Imiglucerase-Sanofi-Genzyme, Velaglucerase-Shire USA, Taliglucerase-Pfizer), MPS I (Aldurazyme – Sanofi – Genzyme), MPS II (Elaprase – Shire USA), Pompe disease (Myozyme- Sanofi Genzyme), Fabry disease (Fabrazyme– Sanofi Genzyme), MPS IV (Vimzim – Biomarin) and MPS VI (Naglazyme (galsulfase)-Biomarin.^60^ There are exorbitant costs associated with these drugs and hence, in India, ERT is provided to LSD patients by companies like Sanofi Genzyme and Takeda Human Genetic Technology as a compassionate access program. In 2017, a total of 240 patients with LSDs received ERT in India through the initiative of various charitable organizations, namely, (i) the INCAP of Sanofi Genzyme; 121 patients with LSDs: Gaucher disease (n = 65), MPS I (n = 20), Fabry disease (n = 13), and Pompe disease (n = 23); (ii) Takeda's HGT's charitable access program; 69 patients with LSDs: Gaucher disease (n = 35), MPS II (n = 27), and Fabry disease (n = 7); and (iii) Protalix; Gaucher disease (n = 5). In addition, 43 children of central and state government organization employees received ERT.^16^ Several factors limit the use of ERT in India. LSD patients on ERT need to have weekly or biweekly intravenous infusions for lifelong. For outstation patients, these visits entail significant travel costs, school absenteeism, and loss of wages for parents.

HSCT is another strategy that has shown success in the treatment of LSDs namely MPS-I, MPS-II, Gaucher disease, MPS VI, MLD, and Krabbe.^61^ However, there is a paucity of data on HSCT in LSDs as stated by Somaraju and Tadepalli in their systematic review.^62^ Also, different aspects of the transplant like donor enzyme level, degree, and persistence of donor chimerism, and post-transplant complications influence the outcomes of BMT and its success.^63^ Literature has shown considerable data on the success of HSCT in MPS I patients. European data has also shown improvement in event-free transplant survival from 58% (1994–2004 data) to 91% (2005–2008 data) for MPS I patients.^64^ Such data is not available at present for MPS II, yet a recent study suggests that HSCT may stabilize neurocognition in MPS II patients when undertaken early in the disease course.^65^ Also, HSCT is now being considered for the treatment of other LSDs like alpha-mannosidosis as studies has shown positive outcomes. The survival rate was 88% in a retrospective study of 17 mannosidosis patients before and after HSCT.^66^ Thus, to assess the risks and benefits of HSCT on different LSDs, additional studies and clinical trials are necessary.

## Prevention of LSDs in India

LSDs are multi-systemic disorders and affect multiple organ systems. Hence, its management involves a cumulative effort from healthcare providers. It requires a multidisciplinary team of haematologists, geneticists, gastroenterologists, paediatricians, neurologists, cardiologists, nephrologists, occupational therapists, and orthopaedic surgeons. There is no approved therapy available for several LSDs and hence in such cases, regular follow-up of patients and providing supportive care are necessary. The fact that LSDs are genetic disorders and have no complete cure; efforts should be directed toward the prevention of this group of conditions. The role of prenatal diagnosis (PND) and genetic counselling is of paramount importance. In India, prenatal diagnostic facilities for LSDs are available for more than two decades^67^ and there are several centres that provide prenatal testing.^68^^,^^69^ Verma et al. conducted PND on 331 subjects and found that about (124/331) 37% were biochemically affected. PND was conducted based on previous affected index cases, confirmed by either enzymatic assay or molecular studies. Prenatal molecular confirmation was done in 43 cases out of 124 biochemically proven and was found to be highly concordant (41/43).^70^ Sheth et al. conducted prenatal diagnosis in 140 pregnancies and observed (60/140) 42.9% to be biochemically affected. PD of LSDs can be carried out by enzyme study from chorionic villi (CV)/cultured CV and cultured amniotic fluid cells (CAF) with an accuracy of the molecular method. However, in cases of MPS and ML-II/III, cultured cells of CVS and AF is preferred over direct CV cells for PD.^69^ In addition, special care is needed while interpreting enzyme results encompassing carrier status and need to be further evaluated by molecular study. In cases where molecular diagnosis has been established in the index case, prenatal diagnosis should be performed by molecular study.

## Management of LSDs in India

### Government initiatives

The Government of India has a crucial leadership role to play in advancing progress in this field to help millions of hopeful and needy patients. In this regard, several initiatives have already taken place in the past five years. In 2014, the Department of Health Research (DHR) and the Indian Council of Medical Research (ICMR) initiated a multicentre collaborative project on research in Lysosomal Storage Disorders with the objective of clinical, biochemical, and molecular characterization of LSDs and studying genotype–phenotype correlation. Table 1 gives details of the data generated by the task force project. The aim was to establish a database of the variants and sequence variations and to establish a smooth network of referral and counselling facilities for affected families across India. The Union Ministry of Health and Family Welfare recently drafted the National Policy for Treatment of Rare Diseases. Two initiatives have been taken that include (i) creating a corpus fund of INR 100 crores for funding the treatment of rare diseases (including LSDs) and (ii) developing a web-based online portal (Crowd funding) for applications for treatment funding. However, there are several challenges faced and our country too can adopt the Singapore model where for every rupee added in Crowd funding, the Government will contribute three rupees against that. This shall develop trust among people who contribute for the cause. Also, a special initiative of a rare disease registry has been taken up recently by the Indian Council of Medical Research to address the incidence and prevalence of different rare diseases across the country. Approximately, 20 centres from across the country are actively involved in the collection and submission of data on LSD and different rare disease cases.

### Patient support groups

About 10 years ago, a non-profit organisation: the Lysosomal Storage Disorders Support Society (LSDDS) was formed by a group of parents of LSD patients and comprising approximately 600 families. LSDDS has been instrumental in bridging the gap between stakeholders, bureaucrats, and physicians. The main aim of LSDDS is to coordinate facilities for the diagnosis and treatment of those suffering from these rare diseases. Recently, individual patients and LSDDS filed writ petitions in the Honourable High Courts in New Delhi, Bengaluru, Chennai, and Hyderabad to direct the government to provide ERT free of cost to patients with LSDs. As an outcome of these litigations, ERT is now being funded for a few patients by the Employee State Insurance Corporation and by the State Government of Karnataka. Organization for Rare Diseases India (ORDI), Indian Organization for Rare disease (IORD), Rare Disease India Foundation (RDIF) are other such patient support groups that cater to the unmet needs of patients suffering from rare diseases in India and also help them for an enrolment in the clinical trials.

### Emerging role of lysosomes

Beyond LSDs, lysosomes seem to have a crucial role as a metabolic command and control centre. It relays multiple nutrient cues to the master growth regulator-mechanistic target of rapamycin complex (mTORC1) kinase that drives de novo lipid synthesis while inhibiting lipid catabolism suggesting its role in nutrient homeostasis.^71^ Since lysosomes also control cellular metabolism and signalling pathways, they play a critical role in treating various diseases including cancer by regulating the maintenance and homeostasis of hematopoietic stem cells, which play an important role in the acute myeloid leukaemia (AML) and other types of cancer.^72^ Recent studies have also shown the link between lysosomal function and Parkinson and Alzheimer's disease due to alpha-synuclein turnover and increase in intracellular level, promoting its accumulation and aggregation.^73^ Lysosomes are also involved in the control of copper-homeostasis. Therefore, besides LSDs, lysosomes have a role in many neurodegenerative diseases, cancer and metabolic disease, and can also serve as a potential therapeutic target.^74^

## Conclusion

Despite all possible efforts and initiatives, LSDs are still underscored in India. Various socio-demographical and economic factors are hurdles in early diagnosis and prevention of LSDs. Moreover, lack of knowledge, awareness and country-wide resources are some factors which contribute to delayed management of this heterogeneous disorders group. For effective tracking and management of LSDs in India, a comprehensive approach and participation from all stakeholders are needed. In addition to government initiatives, the support from non-governmental organisations and channelising the CRS fund may be vital for expanding the services to all corners of the country.

## Contributors

Conceptualisation: J.S., B.J. Review of data and drafting the manuscript: J.S., A.N. and B.J. All the authors reviewed and approved the final version of the manuscript.

## Editor note

The Lancet Group takes a neutral position with respect to territorial claims in published maps and institutional affiliations.

## Declaration of interests

J.S. received research grant from (i) ICMR-DHR-NTF (GIA/31(ii)/2014-DHR) and the funds were used to study the mutation spectrum of different LSDs in the country, (ii) GSBTM-DST/JDR and D/608/2020/459-461 and the funds were used to develop and validate smMIP-based NGS technology for genetic diagnosis of LSD patients, and (iii) BT/PR39587/MED/12/851/2020 and the funds are used to provide genetic diagnosis (detect SNVs and CNVs) in suspected LSD patients using the smMIP-based NGS technology and to develop a national Bio-bank for LSD patients. B.J. co-ordinated the work carried out by the national centers in the DHR-ICMR-NTF LSD project. A.N. is a PhD student working on the development, validation and application smMIP based NGS technology for detecting SNVs and CNVs in suspected LSD patients. There was no funding involved in preparing the current manuscript. The authors declare that the work was conducted in the absence of any commercial or financial relationships that could be construed as a potential conflict of interest.
